# Feedforward Control of Piezoelectric Ceramic Actuators Based on PEA-RNN

**DOI:** 10.3390/s22145387

**Published:** 2022-07-19

**Authors:** Yongcheng Xiong, Wenhong Jia, Limin Zhang, Ying Zhao, Lifang Zheng

**Affiliations:** 1 Shanghai Institute of Applied Physics, Chinese Academy of Sciences, Shanghai 201800, China; xiongyc@mail.ustc.edu.cn (Y.X.); jiawenhong@zjlab.org.cn (W.J.); zhanglimin@zjlab.org.cn (L.Z.); 2 Shanghai Advanced Research Institute, Chinese Academy of Sciences, Shanghai 201204, China; zhaoying@zjlab.org.cn; 3 University of Chinese Academy of Sciences, Beijing 100049, China

**Keywords:** piezoelectric ceramic actuator, hysteretic nonlinearity, recurrent neural network, feedforward control

## Abstract

Multilayer perceptron (MLP) has been demonstrated to implement feedforward control of the piezoelectric actuator (PEA). To further improve the control accuracy of the neural network, reduce the training time, and explore the possibility of online model updating, a novel recurrent neural network named PEA-RNN is established in this paper. PEA-RNN is a three-input, one-output neural network, including one gated recurrent unit (GRU) layer, seven linear layers, and one residual connection in the linear layers. The experimental results show that the displacement linearity error of piezoelectric ceramics reaches 8.96 μm in the open-loop condition. After using PEA-RNN compensation, the maximum displacement error of piezoelectric ceramics is reduced to 0.465 μm at the operating frequency of 10 Hz, which proves that PEA-RNN can accurately compensate piezoelectric ceramics’ dynamic hysteresis nonlinearity. At the same time, the training epochs of PEA-RNN are only 5% of the MLP, and fewer training epochs provide the possibility to realize online updates of the model in the future.

## 1. Introduction

The Shanghai Synchrotron Radiation Facility (SSRF), which operates nearly 5000 h a year, has massive data resources, and the high availability of data brings enormous opportunities for the application of artificial intelligence technology in the field of synchrotron radiation. In recent years, SSRF has explored intelligent beamlines based on the differential evolution algorithm and achieved initial success [1]. However, in the online test, the researchers found that, after the introduction of the second crystal pitch axis of the monochromator [2], the excellent individuals were inherited to the next generation due to its poor motion repeatability, resulting in the intelligent algorithm not converging. SSRF uses many PEAs to achieve micro displacements. However, due to the inherent hysteresis nonlinearity and creep properties, and the defects of the control algorithm, the motion accuracy is not high enough, and the motion repeatability is poor. Studies have shown that, without considering the error caused by vibration, the uncertainty introduced by piezoelectric ceramic hysteresis is generally 15–20%, and the creep error is 1–5% [3].

For a long time, the hysteresis nonlinearity and compensation technology of piezoelectric ceramics have been research hotspots for scholars. The early classical feedforward control technology used mathematical methods to establish a fitting model of the hysteresis loop and to obtain the relationship between the excitation voltage and the actual output displacement by solving its inverse model. Physics-based models and phenomenological models [4] were the main classifications of the hysteresis model in the past. The micro-electromechanical models [5] are typical phenomenological models. Because this classification method is limited, the classifications of rate-independent hysteresis models and rate-dependent hysteresis models according to whether the input rate is considered [6] are more reasonable. Rate-independent hysteresis models include the classic Preisach model [7], the classic Maxwell model [8], and the polynomial model [9]. Furthermore, rate-dependent hysteresis models include the Bouc–Wen mode [10,11] and the micromechanical model [12,13]. Due to the complex nonlinear characteristics, although scholars have proposed many mathematical models, there are certain limitations in the description of the hysteresis loop by various models. Therefore, there has been no widely accepted general model. More and more researchers have introduced other new piezoelectric ceramic feedforward control methods, such as using the Radial basis function(RBF) network [14] and neural network self-turning control [15].

**Figure 1:**
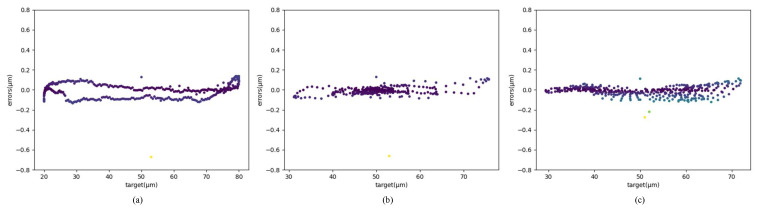
Control flaws of MLP. (**a**) Sinusoidal error distribution. (**b**) Dampened sinusoidal error distribution. (**c**) Amplified sinusoidal error distribution.

MLP has been demonstrated to implement feedforward control of PEA. However, MLP needs too many training epochs, and the control displacement error has jumps and other shortcomings, as shown in Figure 1. Based on the control strategy realized by the MLP, this paper further enriches the piezoelectric ceramic operation dataset and adopts a new network structure(PEA-RNN) that conforms to data characteristics. Accurate displacement output of piezoelectric ceramics is achieved with fewer training epochs. The test results show that, under the operating frequency of 10 Hz, the precision of the controlled precision is improved from 8.96 μm to 0.465 μm after using PEA-RNN compensation. The influence of each network structure (mainly including MLP and residual connection) is verified further to prove the rationality of the network structure.

The rest of this paper is organized as follows: In Section 2, the performance of the experimental platform is evaluated, and the control strategy is preliminarily determined, then the related neural network structures are introduced. Next, the training and the application of PEA-RNN are illustrated, and the effects of MLP and residual connections are verified in Section 3. Finally, this paper is concluded in Section 4.

## 2. Performance Test and Related Structure

### 2.1. Piezoelectric Ceramic Hysteresis Loop

#### 2.1.1. Experimental Platform

**Figure 2:**
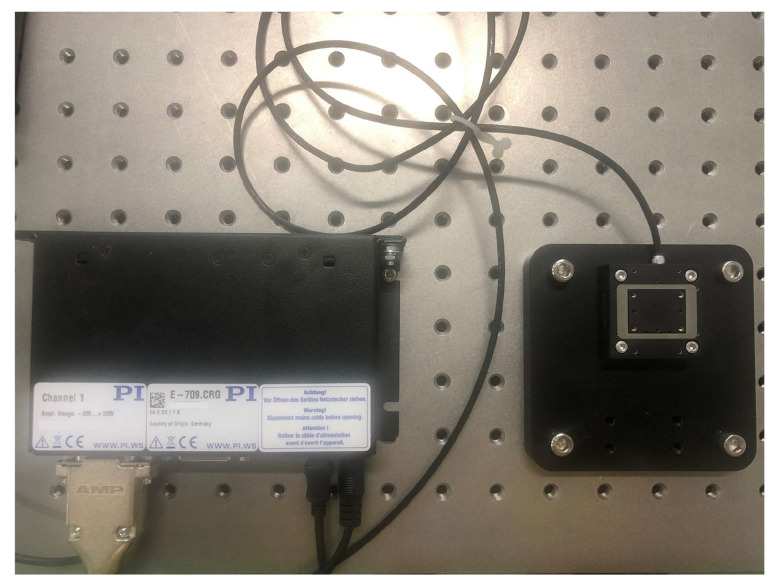
Experimental platform diagram.

**Figure 3:**
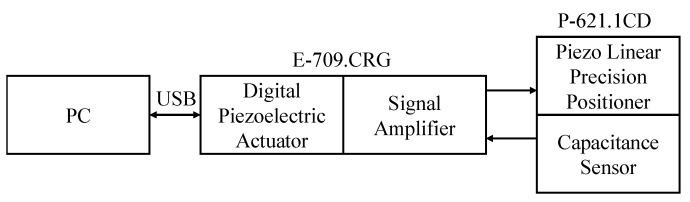
Experimental platform communication diagram.

In this experiment, the digital piezoelectric actuator(E-709.CRG) [16] and the piezo linear precision positioner (P-621.1CD) [17] of Physik Instrumente (PI) are used, as shown in Figure 2. The signal amplifier is integrated into the actuator, and the capacitive sensor is integrated into the positioner. After the actuator receives the command, it sends the voltage to the positioner through the signal amplifier, reads back the current position of the piezoelectric ceramic from the capacitive sensor, and communicates with the computer through USB, as shown in Figure 3.

#### 2.1.2. Primary and Secondary Hysteresis Loops

The hysteresis nonlinearity of piezoelectric ceramics is mainly manifested in input voltage and output displacement curves. The voltage-rising curve does not coincide with the voltage-dropping curve. Many factors affect this characteristic, not only related to the current input voltage, but also related to the input history and the input frequency [18].

The test data were obtained by taking the step signal, the dampened sinusoidal signal, and the amplified sinusoidal signal with a working frequency of 10 Hz as the input. The primary hysteresis loop refers to the largest hysteresis loop formed by the positioner during its entire travel. Furthermore, the secondary hysteresis loop refers to the smaller hysteresis loop.

**Figure 4:**
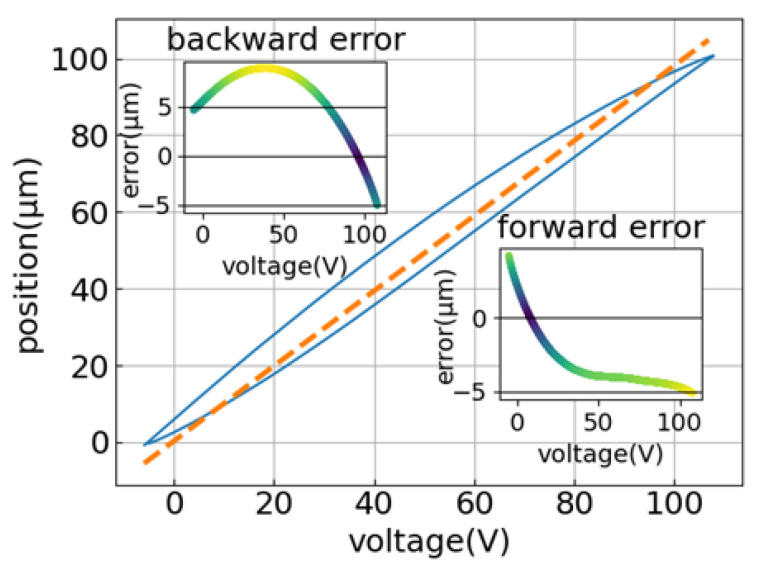
Primary hysteresis loop linear analysis result graph.

As shown in Figure 4, the primary hysteresis loop was linearly fitted, and the linear relationship between the output displacement *x* and the input voltage V was obtained as

(1) x=0.978V+0.294

The maximum deviation of the voltage-rising curve (forward error) is 5.12 μm; the maximum deviation of the voltage-dropping curve (backward error) is 8.96 μm.

**Figure 5:**
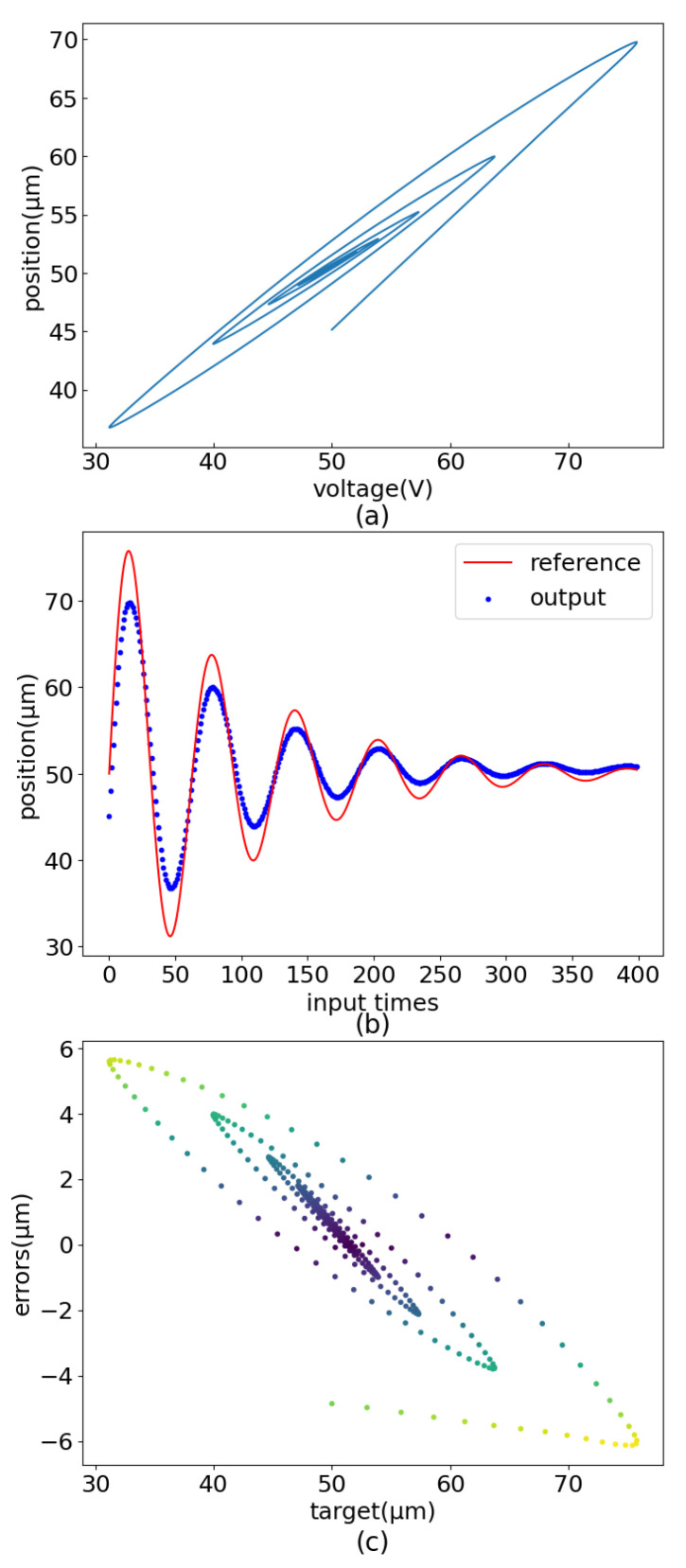
Secondary hysteresis loop linear analysis result graph (dampened sinusoidal input signal). (**a**) Dampened sinusoidal output. (**b**) Dampened sinusoidal curve comparision. (**c**) Dampened sinusoidal error distribution.

**Figure 6:**
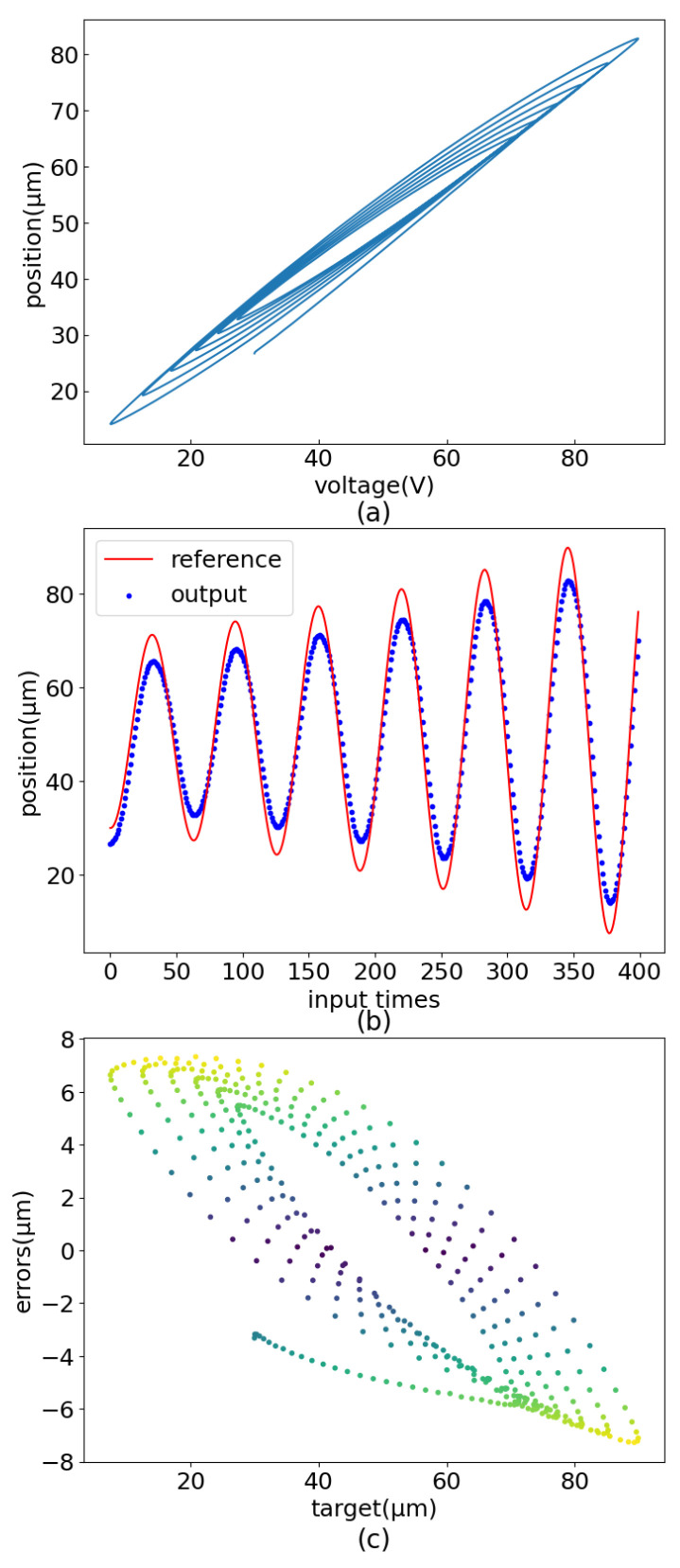
Secondary hysteresis loop linear analysis result graph (amplified sinusoidal input signal). (**a**) Amplified sinusoidal output. (**b**) Amplified sinusoidal curve comparision. (**c**) Amplified sinusoidal error distribution.

The test results of the secondary hysteresis loop are shown in Figure 5 and Figure 6, and the maximum errors in the open loop are all about 6 μm.

#### 2.1.3. Hysteresis Loop Model

It can be found from the primary and the secondary hysteresis loops that the current position of the positioner has a great relationship with the current output voltage, the last position, and the last input voltage.

The state $\Phi_{t}$ of the piezoelectric ceramic at time t is uniquely represented by the input voltage and output displacement at the current time.

(2) $$\Phi_{t}=\varphi \left(V_{t},x_{t}\right)$$

where $V_{t}$ is the input voltage at time t, and $x_{t}$ is the displacement at time t.

From the diagrams of the primary and the secondary hysteresis loops, it is not difficult to see the relationship between the current state and the historical state, and there is an obvious time series relationship between $\Phi_{1}$, ⋯, $\Phi_{t}$, which can be represented by a conditional probability model.

(3) $$P\left(\Phi_{1},\cdots ,\Phi_{t}\right)=\prod_{T=1}^{t}P\left(\Phi_{T}|\Phi_{1},\cdots ,\Phi_{T-1}\right)\;\mathrm{where}\;P\left(\Phi_{1}|\Phi_{0}\right)=P\left(\Phi_{1}\right)$$

As the operating time of PEA increases, more and more piezoelectric ceramic states need to be recorded and calculated, which will directly lead to an increasing control time and a continuous decrease in operating frequency. Therefore, consider introducing the Markov assumption, suppose that in the real situation, the rather long sequence $\Phi_{t-1}$, …, $\Phi_{1}$, may not be necessary for the current state $\Phi_{t}$. Therefore, it is only necessary to satisfy a certain length τ; using the observation sequence $\Phi_{t-1}$, …, $\Phi_{t-\tau }$, the current state can be predicted. According to the Markov assumption, (3) can be modified as

(4) $$P\left(\Phi_{1},\cdots ,\Phi_{t}\right)=\prod_{T=1}^{t}P\left(\Phi_{T}|\Phi_{T-\tau },\cdots ,\Phi_{T-1}\right)$$

To meet the requirements of high response frequency, a simple first-order Markov model is used, that is, τ=1. (4) can be simplified as

(5) $$P\left(\Phi_{1},\cdots ,\Phi_{t}\right)=\prod_{T=1}^{t}P\left(\Phi_{T}|\Phi_{T-1}\right)\;\mathrm{where}\;P\left(\Phi_{1}|\Phi_{0}\right)=P\left(\Phi_{1}\right)$$

Bringing (2) into the equation

(6) $$x_{t}=f\left(V_{t},x_{t-1},V_{t-1}\right)$$

$V_{t-1}$ is the input voltage at time t−1, and $x_{t-1}$ is the displacement at time t−1.

After transformation, (7) is obtained:

(7) $$V_{t}=f^{\prime }\left(x_{t},x_{t-1},V_{t-1}\right)$$

Finally, modifying the parameters:

(8) $$V_{R}=f^{\prime }\left(x_{T},x_{t-1},V_{t-1}\right)$$

For the piezoelectric ceramic positioner, from the given target position $x_{T}$, the last time input voltage $V_{t-1}$ and the last time displacement $x_{t-1}$, the voltage applied to the positioner $V_{R}$ is calculated by the function $f^{\prime }$.

**Figure 7:**
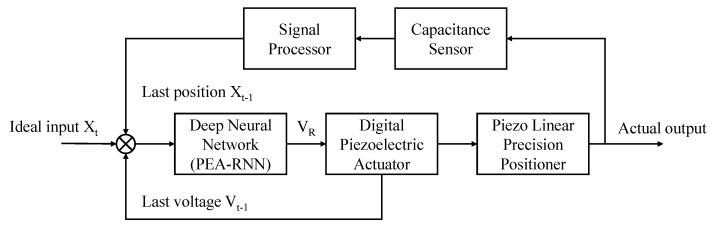
Schematic diagram of feedforward control of PEA-RNN.

Figure 7 is the principle structure diagram of the whole system. The task of this paper is to design a feedforward control model of a recurrent neural network, perform compensation calculation on the preset target position, and output the result through the controller to apply it to piezoelectric ceramics to realize micro and precise displacement.

### 2.2. Network Structure

#### 2.2.1. Recurrent Neural Network (RNN) and Gated Recurrent Unit (GRU)

In the model, considering the state of the last moment as a hidden variable for processing, it is necessary to add a neural network structure that can describe the hidden variable, which is the RNN [19].

**Figure 8:**
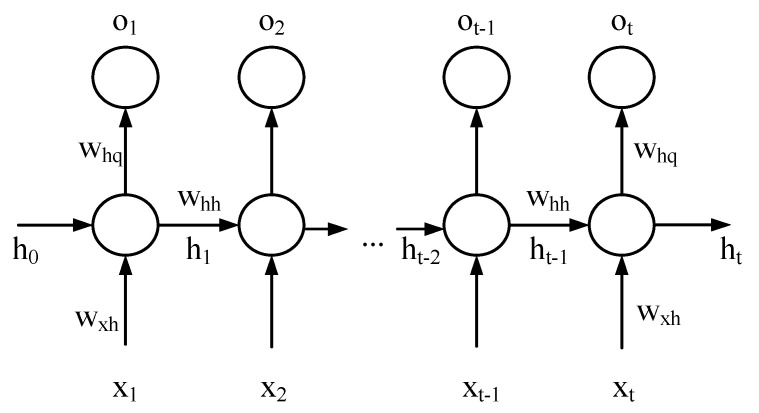
Recurrent Neural Unit Computational Flowchart.

As shown in Figure 8, for a single recurrent neuron, the input $x_{t}$, at time t, needs to be calculated in three steps to obtain the output $o_{t}$ (to simplify the description, the input layer bias and the output layer bias are ignored). Firstly, the input layer weight $w_{xh}$ is multiplied; secondly, the hidden layer output $h_{t-1}$, at time t−1, is multiplied by the weight $w_{hh}$, and then added to the first step result to obtain $h_{t}$; thirdly, $h_{t}$ is multiplied by the output layer weight $w_{hq}$, activated by the activation function, and the final output is $o_{t}$.

The first step is usually expressed together with the second step:

(9) $$h_{t}=w_{\mathrm{xh}}x_{t}+w_{\mathrm{hh}}h_{t-1}$$

The third step can be expressed as:

(10) $$o_{t}=\sigma \left(w_{\mathrm{hq}}h_{t}\right)$$

The hidden layer state h is continuously transmitted and used “recurrently”, which coincides with the time sequence characteristic of the piezoelectric ceramic operating data.

The structure of the basic recurrent neural unit is simple, but for long sequence problems, the training likely fails due to gradient disappearance or explosion. GRU is a recurrent neural unit with better performance optimized for the shortcomings of the basic one [19].

**Figure 9:**
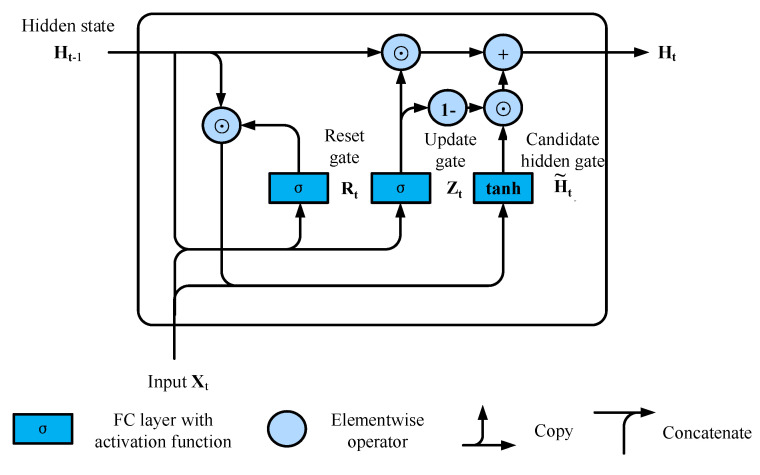
GRU computational flow chart.

The calculation flow of the GRU is shown in Figure 9. GRU mainly includes the reset gate and the update gate, which are vectors in the interval (0,1). The opening of the reset gate controls how well the current input combines with the hidden state, while the opening of the update gate controls the retention of the hidden state [20].

For a given time step t, assuming the input is a mini-batch $X_{t}\in R^{n\times d}$ (number of samples: n, number of inputs: d), the hidden state of the previous time step is $H_{t-1}\in R^{n\times h}$ (number of hidden units: h). Then the reset gate $R_{t}\in R^{n\times h}$ and the update gate $Z_{t}\in R^{n\times h}$ are calculated as follows:

(11) $$R_{t}=\sigma \left(X_{t}W_{xr}+H_{t-1}W_{hr}+b_{r})\right.$$

(12) $$Z_{t}=\sigma \left(X_{t}W_{xz}+H_{t-1}W_{hz}+b_{z})\right.$$

where σ represents the sigmoid function, which is used to convert the input value to the interval (0,1), the expression is

(13) $$\mathrm{sigmoid}\left(x\right)=\frac{1}{1+e^{-x}}$$

Next, the reset gate $R_{t}$ is integrated with the regular hidden state update mechanism to obtain candidate hidden states ${\tilde{H}}_{t}\in R^{n\times h}$ at time step t

(14) $${\tilde{H}}_{t}=\tanh\left(X_{t}W_{\mathrm{xh}}\right.+\left(R_{t}⊙H_{t-1}\right)W_{hh}\left.+b_{h}\right)$$

where the symbol ⊙ is the Hadamard product operator. The role of the tanh function is to convert the input value to the interval (−1,1); the specific expression is

(15) $$\tanh\left(x\right)=\frac{1-e^{-2x}}{1+e^{-2x}}$$

The result of the calculation is the candidate because the operation of the update gate still needs to be combined. The element multiplication of $R_{t}$ and $H_{t-1}$ in (14) can reduce the influence of past states. Whenever the terms in the reset gate $R_{t}$ approach 1, restore a normal recurrent neural network. For all terms close to 0 in $R_{t}$, the candidate hidden state is the result of an MLP with $X_{t}$ as input. Therefore, any pre-existing hidden state is reset to default. Finally, the effect of the update gate $Z_{t}$ needs to be combined, which determines to what extent the new hidden state $H_{t}$ is the old state $H_{t-1}$, and the use of the new candidate state ${\tilde{H}}_{t}$ quantity. The update gate $Z_{t}$ only needs an element-wise convex combination between $H_{t-1}$ and ${\tilde{H}}_{t}$ to achieve this. This provides the final update formula for the gated recurrent unit:

(16) $$H_{t}=Z_{t}⊙H_{t-1}+\left(1-Z_{t}\right)$$

Whenever the update gate $Z_{t}$ approaches 1, only the old state is kept. At this point, the information from $X_{t}$ is essentially ignored, effectively skipping time step t in the dependency chain. Conversely, as $Z_{t}$ approaches 0, the new hidden state $H_{t}$ approaches the candidate hidden state ${\tilde{H}}_{t}$. These designs can help deal with the vanishing gradient problem in recurrent neural networks and better capture the dependencies of sequences with long time-step distances. For example, if the update gate is close to 1 for all time steps of the entire subsequence, then regardless of the length of the sequence, the old hidden state at the beginning time step will be easily preserved and passed on to the end of the sequence.

In summary, GRU has the following two salient features: [19]

1. Reset gate helps capture short-term dependencies in the sequence;
2. Update gate helps capture long-term dependencies in the sequence.

#### 2.2.2. Multilayer Perceptron (MLP)

**Figure 10:**
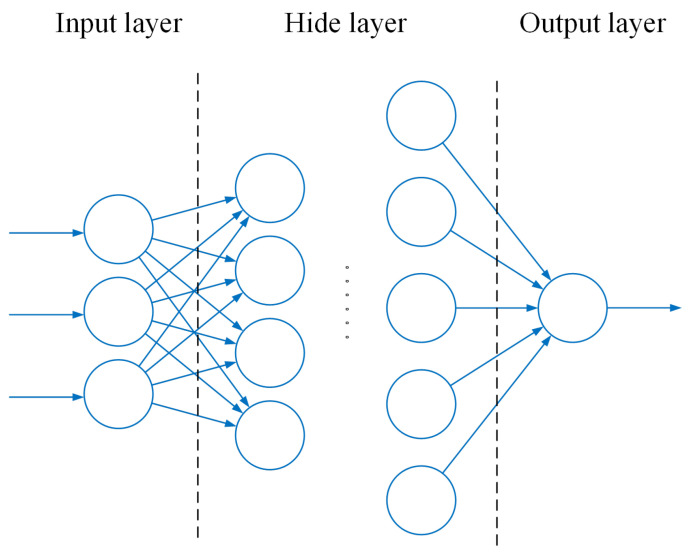
MLP structure diagram.

The structure of MLP is shown in Figure 10. This structure can improve the performance of the network and realize dimensional transformation.

#### 2.2.3. Residual Connection

Generally, increasing the number of layers can improve the neural network performance, but it will correspondingly increase the computing time and the resources occupied by the calculation. At the same time, the increase in the number of layers will also lead to network degradation—that is, the weight matrix in the network will no longer be sensitive to changes in the input in some dimensions, and the same output is obtained regardless of the input. To optimize the MLP, the number of layers should be compressed as much as possible without loss of performance or very little performance, so the residual connection is introduced into MLP in the experiment.

**Figure 11:**
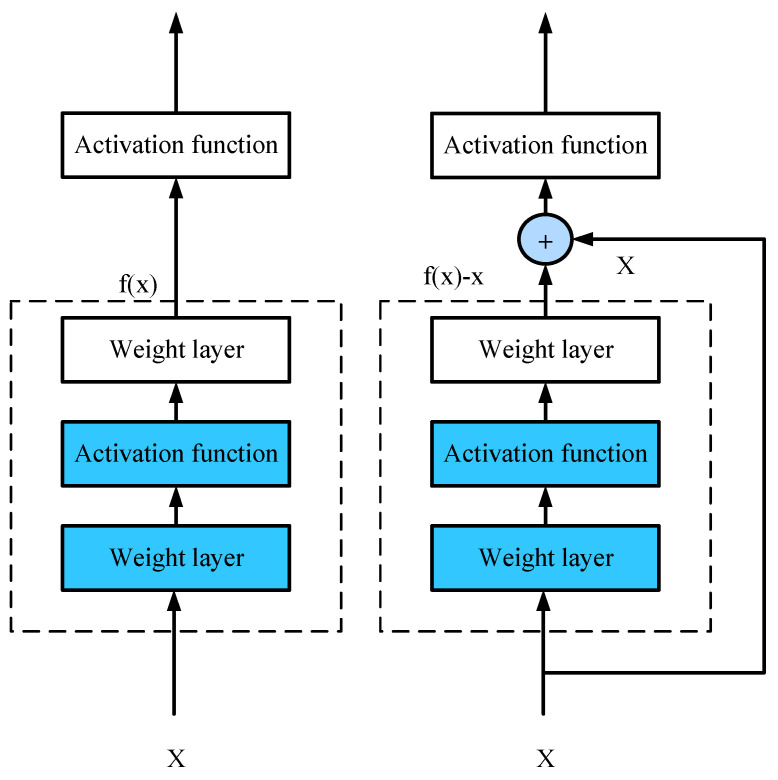
Residual connection structure diagram.

Focus on the network portion: As shown in Figure 11, suppose the original input is x, and the ideal map you want to train is f(x) (as input to the activation function above). The part in the dashed box on the left needs to fit the mapping f(x) directly, while the part in the dashed box on the right needs to fit the residual mapping f(x)-x. Residual maps tend to be easier to optimize in reality. The right picture is the basic structure of ResNet [21]—the residual block. Using the residual connection can break the symmetry of the neural network, making the input propagate forward faster between layers, and more conducive to constructing deep neural networks.

## 3. Training and Application of PEA-RNN

### 3.1. Network Training

**Figure 12:**
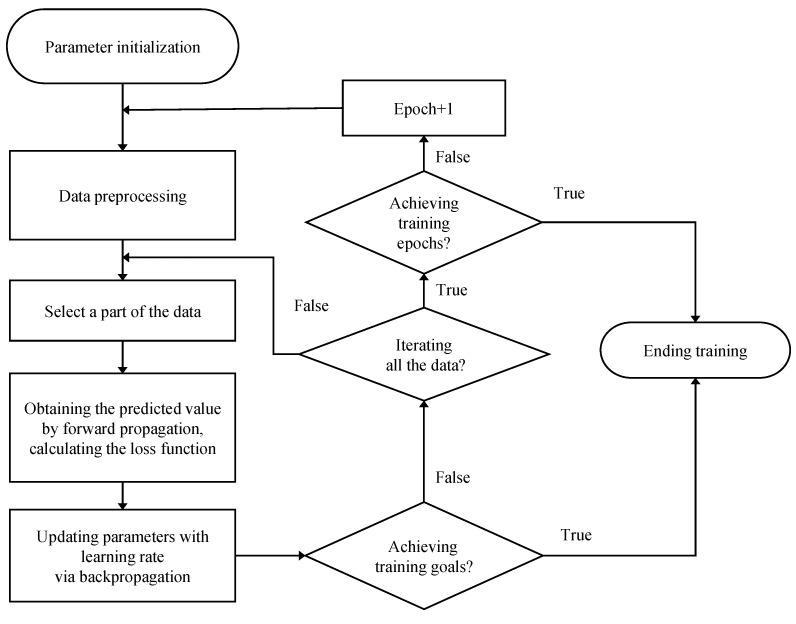
Neural network training process flow chart.

The training process is shown in Figure 12. Firstly, initialize the weight value parameters, scramble the dataset randomly, and divide it into a training dataset and a verification dataset according to the ratio of 8:2. Then, take out the training data in groups, take the current actual displacement, the last time voltage and the last time displacement as the input of the neural network, set the initial state to all zeros, and calculate the voltage prediction layer by layer through the forward propagation algorithm. After getting the prediction, compare it with the current actual voltage and calculate the loss value (mean square error, MSE). The neural network weight parameters are updated according to the optimizer in the backpropagation algorithm. Whenever the loss value on the training dataset decreases, the network is verified using the verification dataset. If the loss value is the minimum loss value on the verification dataset at this time, the current neural network parameters are saved.

In the experiment, the loss function used is the MSE function, and the specific expression is:

(17) $$\mathrm{MSE}=\frac{1}{n}\sum_{i=1}^{n}{\left(y_{i}-{\hat{y}}_{i}\right)}^{2}$$

where *n* represents the number of samples.

The optimizer uses the most commonly Adam [22], which can adaptively adjust the learning rate α so that the training can converge faster. The parameter update formula is:

(18) $$w_{t}\leftarrow w_{t-1}-\alpha_{t}\cdot m_{t}/\left(\sqrt{v_{t}}+\hat{\epsilon }\right)$$

In the formula, $w_{t}$ is the weight parameter to be updated; $w_{t-1}$ is the weight parameter at the last moment; $\alpha_{t}$ is the learning rate, and $m_{t}$ is the first-order moment estimation value of the gradient, which can adaptively adjust the speed of the learning rate change; $v_{t}$ is the second-order moment estimate of the gradient, which can prevent the parameter from falling into a local minimum; $\hat{\epsilon }=10^{-8}$, which is used to prevent the divisor from being 0.

**Figure 13:**
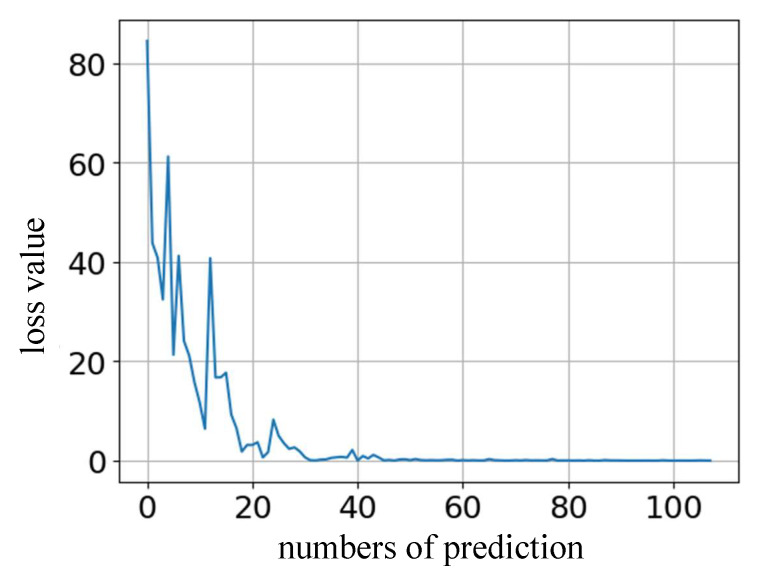
RNN Prediction Error Curve.

The total dataset comprises the piezoelectric ceramic operating data, with 16,020 sets. During the experiment, set the Batch Size to 32 and the learning rate to 0.01. After 5000 epochs of training, a model that meets the accuracy requirements is obtained. The error decrease on the validation dataset is shown in Figure 13.

### 3.2. Network Application

#### 3.2.1. The Overall Structure of PEA-RNN

**Figure 14:**
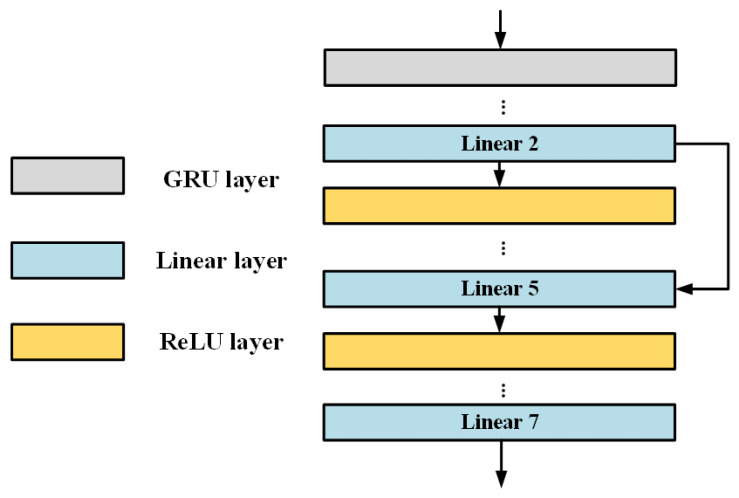
PEA-RNN Structure Diagram.

As shown in Figure 14 and Table 1, the designed deep neural network includes one recurrent layer (hidden state dimension is 128) and seven linear layers. The ReLU function is used as the activation function between the linear layers, and the second layer of the linear layer connects with the fifth layer by Residual connection. The input layer contains three dimensions (current actual displacement, the last time voltage, and the last time displacement), and the output layer is one-dimensional (input voltage prediction).

According to the design, for the ideal input voltage $V_{k}$ required to move to the target displacement $x_{k}$ at time k, the operation process is as follows:

Step 1: The target displacement $x_{k}$, the output displacement $x_{k-1}$ and the input voltage $V_{k-1}$ at time k−1 directly form the input matrix $X^{\left(k\right)}$($X^{\left(k\right)}\in R^{1\times 3}$) of the neural network at time k, and set the initial hidden state matrix $H_{0}^{\left(k\right)}$($H_{0}^{\left(k\right)}\in R^{1\times 128}$) to zero:

(19) $$X^{\left(k\right)}=\left[\begin{array}{ccc} x_{k-1} & V_{k-1} & x_{k} \end{array}\right]$$

(20) $${H_{0}}^{k}=0$$

Step 2: GRU processes the input matrix sequentially and then outputs the corresponding result and the hidden state matrix containing trend information.

(21) $$\begin{array}{c} H_{1}^{\left(k\right)}=Z_{0}^{\left(k\right)}⊙H_{0}^{\left(k\right)}+\left(1-Z_{0}^{\left(k\right)}\right)⊙{\tilde{H}}_{0}^{\left(k\right)} \end{array}$$

(22) $$\begin{array}{c} H_{2}^{\left(k\right)}=Z_{1}^{\left(k\right)}⊙H_{1}^{\left(k\right)}+\left(1-Z_{1}^{\left(k\right)}\right)⊙{\tilde{H}}_{1}^{\left(k\right)} \end{array}$$

(23) $$\begin{array}{c} H_{3}^{\left(k\right)}=Z_{2}^{\left(k\right)}⊙H_{2}^{\left(k\right)}+\left(1-Z_{2}^{\left(k\right)}\right)⊙{\tilde{H}}_{2}^{\left(k\right)} \end{array}$$

Step 3: The output result $H_{3}^{\left(k\right)}$ of GRU reduces the dimension from 128 to 3 through a linear layer, and obtains the operation result of the recurrent layer $O_{\mathrm{RNN}}^{\left(k\right)}$($O_{\mathrm{RNN}}^{\left(k\right)}\in R^{1\times 3}$).

Step 4: The operation result of the recurrent layer $O_{\mathrm{RNN}}^{\left(k\right)}$ continues to be used as the input of the MLP $X_{\mathrm{MLP}}^{\left(k\right)}$. After the calculation of the MLP, the dimension is reduced from 3 to 1, This result is the ideal input voltage $V_{k}$ at time k.

(24) $$X_{\mathrm{MLP}}^{\left(k\right)}=O_{\mathrm{RNN}}^{\left(k\right)}$$

(25) $$V_{k}=F\left(X_{\mathrm{MLP}}^{\left(k\right)}\right)$$

where F(·) represents the complex nonlinear computation constructed by the MLP.

After the calculation of the recurrent layer and the MLP, the ideal input voltage $V_{k}$ of the piezoelectric driver is finally obtained.

(26) $$V_{k}=f^{\prime }\left(x_{k},x_{k-1},V_{k-1}\right)$$

#### 3.2.2. Experimental Test

**Figure 15:**
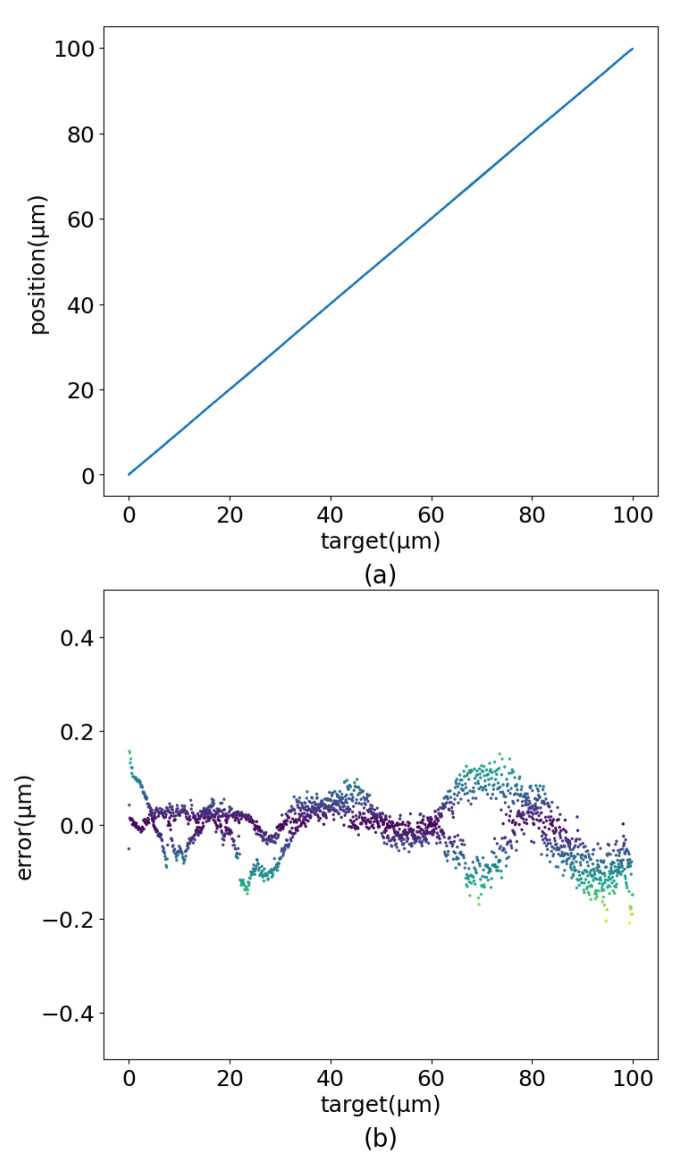
Linear motion control renderings. (**a**) Output. (**b**) Error.

According to Figure 15, the validity of the model was verified on the experimental platform (Figure 2). Set the target position on the PC to change with the linear motion trajectory, sinusoidal motion trajectory, amplified sinusoidal motion trajectory, and damped sinusoidal motion trajectory with a working frequency of 10 Hz. After PEA-RNN feedforward compensation, the prediction is sent to the controller to generate input voltage. Finally, test the actual position of the positioner.

**Figure 16:**
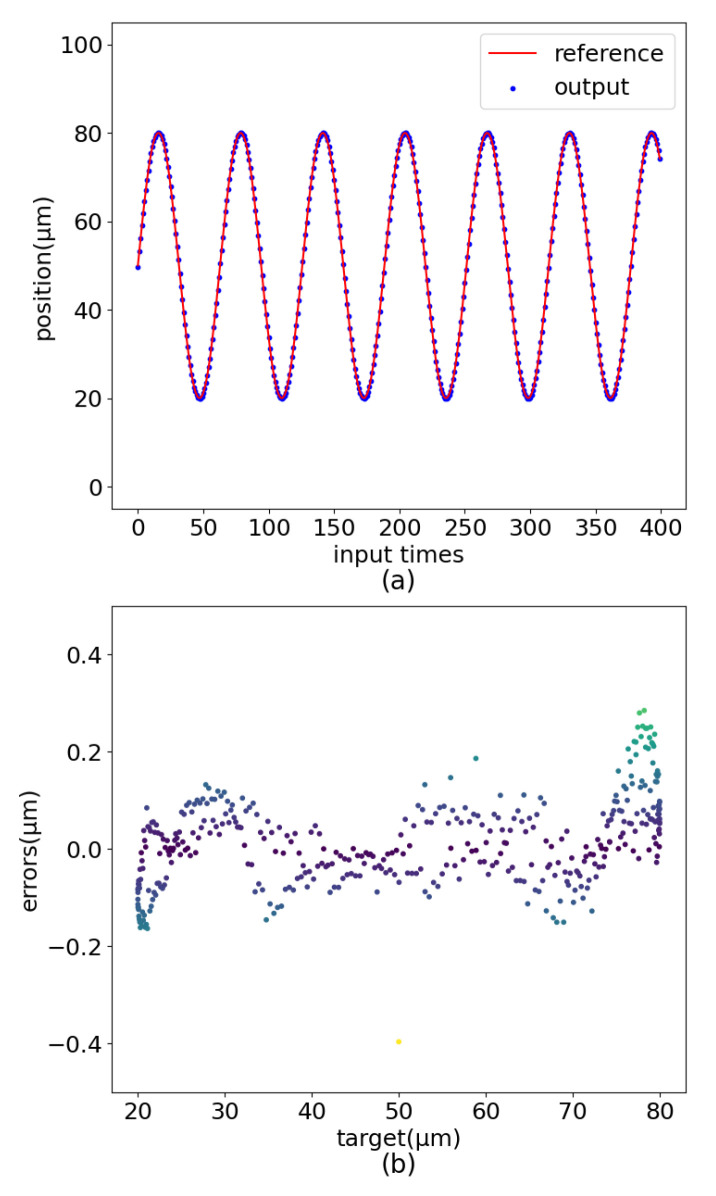
Sinusoidal motion control renderings. (**a**) Curve comparision. (**b**) Error distribution.

**Figure 17:**
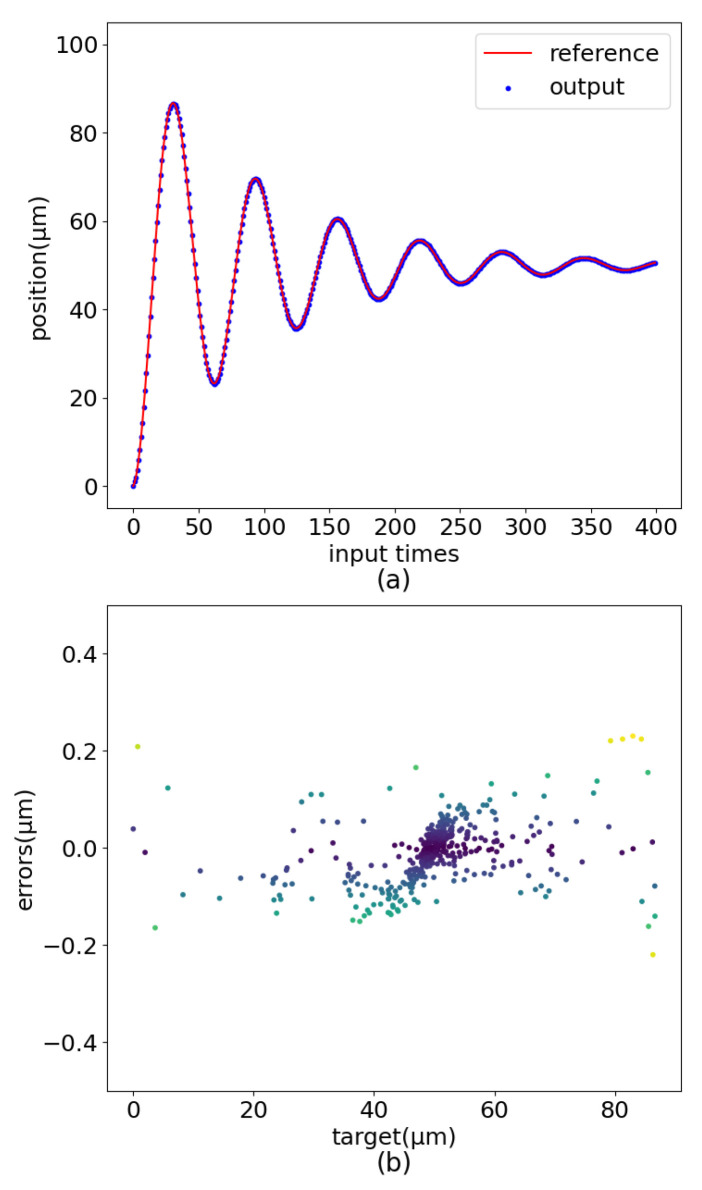
Dampened Sinusoidal motion control renderings. (**a**) Curve comparision. (**b**) Error distribution.

**Figure 18:**
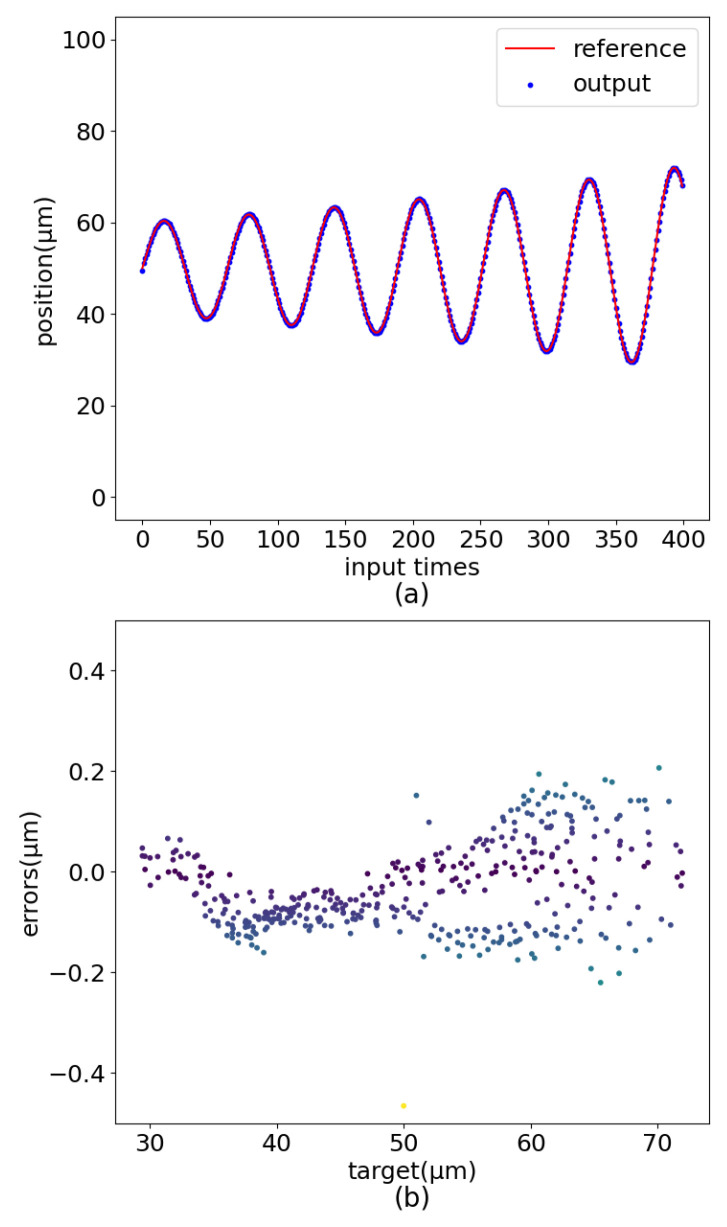
Amplified Sinusoidal motion control renderings. (**a**) Curve comparision. (**b**) Error distribution.

The experimental results are shown in Figure 16, Figure 17 and Figure 18. When set as the step signal, the target position has a good linear relationship with the actual position, and the maximum error value is 0.210 μm. When the target is set to a sinusoidal signal, the maximum error is 0.396 μm. When the target is set to a dampened sinusoidal signal, the maximum error is 0.230 μm. The maximum error is 0.465 μm when the target is set to an amplified sinusoidal signal.

### 3.3. Ablation Experiment

To more clearly demonstrate the contribution of each structure in the PEA-RNN to the model, two ablation experiments were performed to evaluate the effect of the MLP and the residual connection, respectively.

#### 3.3.1. The Impact of MLP

**Figure 19:**
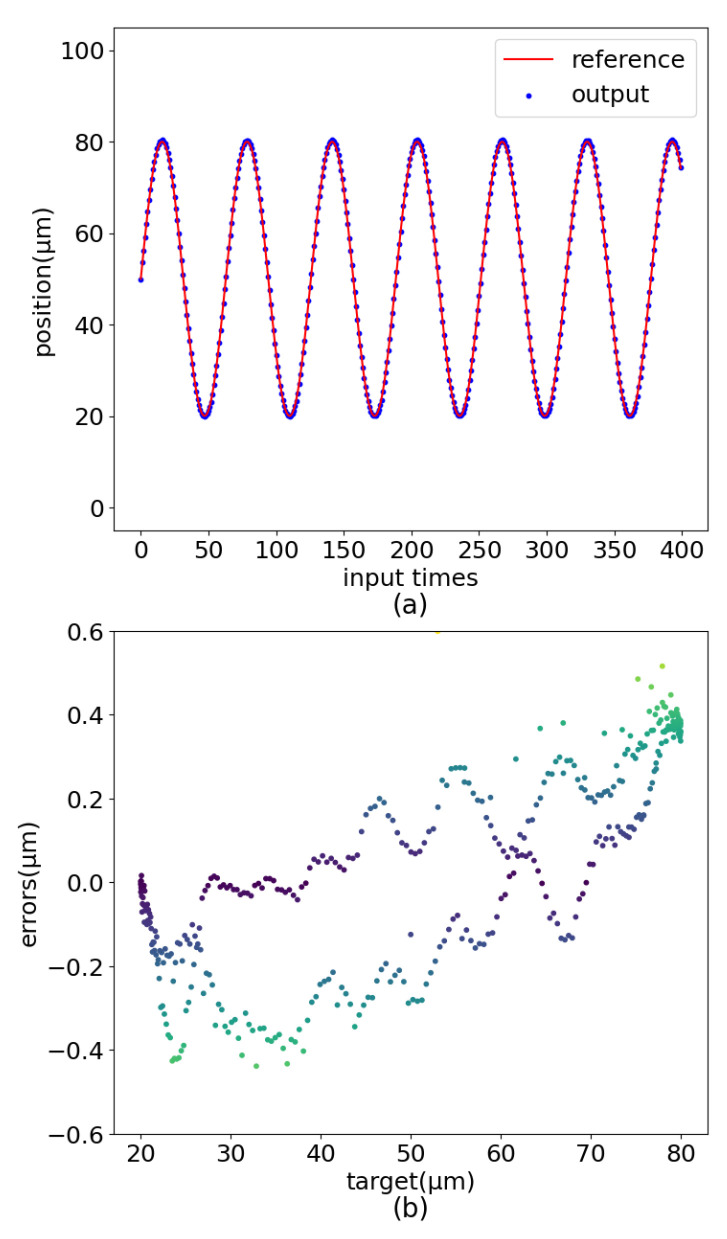
PEA-RNN without MLP control errors. (**a**) Curve comparision. (**b**) Error distribution.

This experiment aims to verify the effect of MLP on the control accuracy of PEA-RNN. The experimental method adopted is to directly delete the MLP in PEA-RNN, so the new network has only one GRU layer. After the network is fully trained with the same training strategy as the original, the output error of the piezoelectric ceramic is tested under the condition that the input is a sinusoidal signal. The results are shown in Figure 19. The maximum output error is 0.599 μm, an increase of 51.3% compared to the original network.

After removing the MLP, the new network is a normal RNN, including a GRU layer. The linear fitting ability of the network is reduced, the error distribution is also relatively discrete, and the error at the starting point is significant. The MLP at the tail can enhance the nonlinear expression ability of PEA-RNN so that the model can describe the hysteresis nonlinearity of piezoelectric ceramics in appropriate detail.

#### 3.3.2. The Impact of Residual Connection

**Figure 20:**
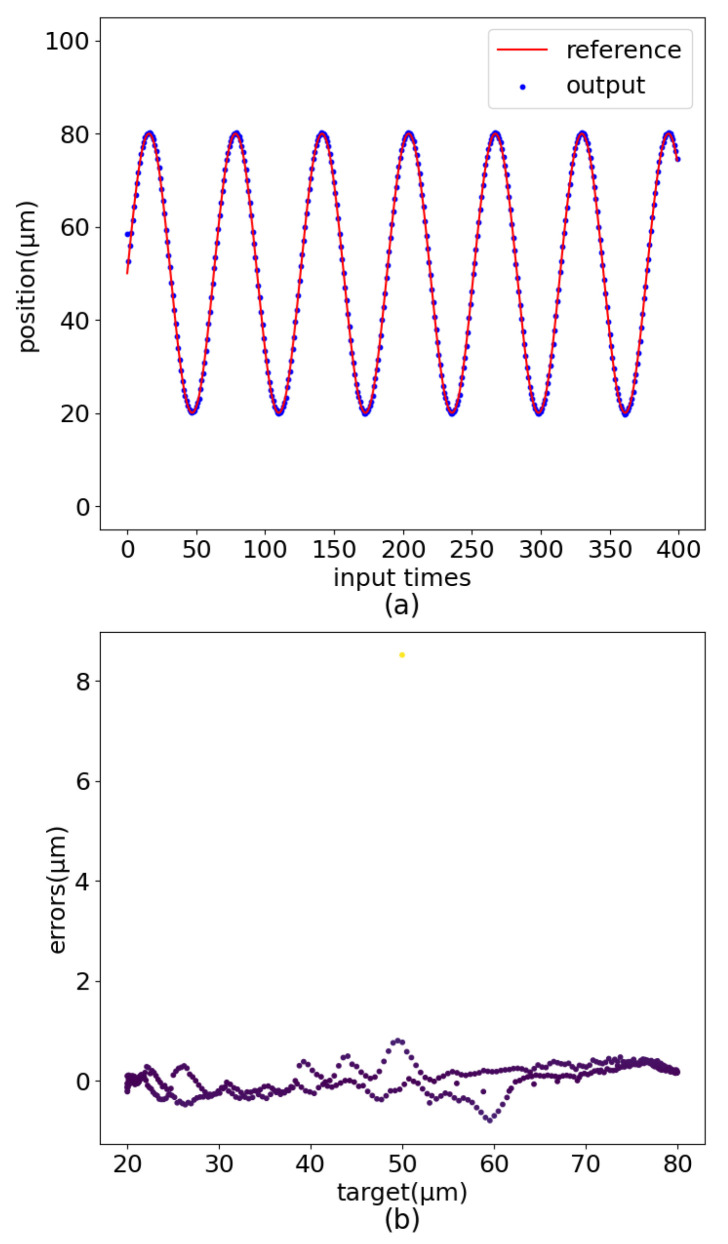
PEA-RNN without Residual Connection control errors. (**a**) Curve comparision. (**b**) Error distribution.

This section evaluates its impact on model performance by removing the residual connection in PEA-RNN alone, and the experimental test results are shown in Figure 20. At the beginning of the piezoelectric ceramic displacement output, the error is too large, indicating that the neural network has not completely fitted the original curve after the same epochs of training, and the training error has not dropped to a relative minimum. A part of the error value is around ±1 μm, which is far worse than the original network control accuracy.

The role of the residual connection is to speed up the neural network training process. At the same time, it is beneficial to build a deeper network, which can also increase the nonlinear expression ability of the network.

## 4. Conclusions

Based on the PyTorch framework, this paper builds a deep neural network model named PEA-RNN, with one recurrent layer and seven linear layers. The ReLU function is used as the activation function between the linear layers. The residual connection is used between the second and fifth layers of the linear layers. The model’s design principle and training process is given, and the model obtained by training is applied to the feedforward compensation of the positioner.

The test results show that the maximum displacement error is reduced from 8.96 μm to 0.465 μm under the control of PEA-RNN with the input of the 10 Hz operating frequency. Through ablation experiments to verify the role of each structure in the PEA-RNN, MLP can effectively enhance the nonlinear expression ability of the model, and the residual connection can not only accelerate the training process but also enhance the nonlinear expression of the model. These results show that PEA-RNN constructed in this paper can accurately describe the dynamic hysteresis nonlinearity of piezoelectric ceramics, realize the real-time intelligent beam line modulation system, and create the possibility for online updating of the model.

## Figures and Tables

**Table 1:** RNN Specific Structure Parameters.

Layer Name	Input Dimension	Output Dimension	Bias	Hidden State Dimension
GRU layer	3	3	True	128
Linear 1	3	16	True	/
Linear 2	16	32	True	/
Linear 3	32	64	True	/
Linear 4	64	128	True	/
Linear 5	128	32	True	/
Linear 6	32	4	True	/
Linear 7	4	1	True	/

## Abbreviations

The following abbreviations are used in this manuscript:

MLP	Multilayer perceptron
PEA	Piezoelectric actuator
RBF	Radial basis function
GRU	Gated recurrent unit
SSRF	Shanghai Synchrotron Radiation Facility
PI	Physik Instrumente
MSE	Mean square error
ReLU	Linear rectification function
